# Development of Thai Sensory Patterns Assessment Tool for Children Aged 3–12 Years: Caregiver-Version

**DOI:** 10.3390/healthcare10101968

**Published:** 2022-10-08

**Authors:** Revadee Sutthachai, Anuchart Kaunnil, Supaluck Phadsri, Ilada Pomngen, Mandy Stanley, Tiam Srikhamjak

**Affiliations:** 1 Department of Occupational Therapy, Faculty of Associated Medical Sciences, Chiang Mai University, Chiang Mai 50200, Thailand; 2 School of Medical and Health Sciences, Edith Cowan University, Joondalup, WA 6027, Australia

**Keywords:** sensory processing patterns, sensory preference, sensory arousal, sensory assessment, validity, reliability, health promotion assessment, children

## Abstract

Most existing tools for measuring sensory patterns of children have been developed in Western countries. These tools are complex and may not be culturally appropriate for other contexts that require specific knowledge in the clinical perspective. The aim of this study was to develop a simplified tool called the Thai Sensory Patterns Assessment (TSPA) tool for children. It is designed for children ages 3–12 years old to be completed by their caregiver. The process of creating the tool consisted of drafting a questionnaire and interpreting the result. Partial psychometrics were completed during item development, content validity of items was assessed by five expert ratings. Construct validity and internal consistency were assessed using data from 414 caregivers and intra-rater reliability was assessed with 40 caregivers. The two parts of the TSPA tool for children results, sensory preference, and sensory arousal, were designed to be presented as a sensory pattern in a radar chart/plot. The data analysis showed that both parts of the TSPA tool for children had acceptable psychometric properties with the retained 65 items. Only proprioceptive sensory arousal had a low Cronbach’s α coefficient, suggesting more information sharing between caregivers and professionals is needed. This research is an initial study and must be continuously developed. Future development of this tool in technology platforms is recommended to support use within healthcare services.

## 1. Introduction

Sensory processing refers to the reception, modulation, integration, and organization of sensory stimuli as well as behavioral response to sensory input [1,2]. Sensory processing is the building blocks of perception, emotion, behavior, and development. Research studies in sensory processing investigate that individuals have different patterns of processing that can be observed behaviorally and neurophysiological [3,4]. Differential sensory patterns can be associated with human characteristics. Several previous studies indicated that sensory processing patterns may influence human behavior at all ages. The study in adulthood found that high and low external stimulation can be distinguished are related to higher fatigue frequencies in adulthood [5]. In addition, the study found that a calm atmosphere important strategy to control agitation, as well as sensory defensiveness in adults, which may be a tendency towards increased symptoms of anxiety and depression [6,7]. It is also studied in adolescents to demonstrate that adolescents’ sensory avoidance may be related to pain experience, pain catastrophizing, and disability level [8]. Especially, children are one of the most important periods of human development, and sensory stimuli are most effective for them. Not only does sensory processing affect daily routine activities, social, cognitive, and sensorimotor development in children but also health and illness. For example, the study found that sensory processing patterns relate to conduct problems and inattentive and hyperactive behavior. It is also sensory processing patterns factors were significantly associated with the children’s sleep patterns [9,10]. Furthermore, the study found that sensory processing patterns and children’s traumatic experiences may specifically characterize individuals with affective disorders and prediction of their quality of life [11].

Currently, many sensory processing pattern assessments are used for the implementation of healthcare services. However, the most of previous existing tools are designed for healthcare professionals or therapists such as occupational therapists [12,13,14]. Furthermore, it required more specific knowledge in the clinical context or specific knowledge. Sensory processing issues are well known in the medical context but rarely in general people include caregivers of children. In Thailand, Tiam Srikhamjak and colleagues developed the Thai Sensory Patterns Assessment (TSPA) tool for assessing sensory processing patterns [15,16,17,18]. The tool was developed by modifying Dunn’s sensory profile toward Thai adolescents and adults and developed continuously for more than 15 years. There are two parts of the TSPA tool for adolescents and adults, sensory preferences, and sensory arousal. Sensory preference is defined as the behavioral expression preferred in a particular sensory stimulus in daily life. Sensory arousal is defined as a behavioral expression that responds impulsively to a specific sensory stimulus in daily life. It is interesting that the TSPA tool is simple to use and can be used to link behavior responses to sensory stimuli in a variety of ways. The results are presented in picture form with each sensory domain specified and interpreted in simple layman’s terms. Further, Thai research indicates that the TSPA tool for adolescents and adults can provide a feasible tool for identifying sensory preferences to match health promotion modalities appropriately. The previous study classified the level of the participants’ sensory preferences concerning their cortisol levels by using Mindfulness-Based Flow Practice (MBFP) [19]. It is also a TSPA tool that is used to evaluate the process for planning healthcare services in clinical and community settings. As this tool is easy to use, it has been put to a variety of uses.

In Thailand, healthcare providers and health volunteers work with family caregivers. To enhance caregivers understanding of their children, our team is developing a simple tool. It enables family caregivers to improve the health and well-being of their children based on the results of an assessment tool. In Thailand, an assessment of sensory patterns was carried out, but the tool was intended for adults and adolescent. For children (caregiver version), there is no simple tool for sensory patterns assessment. To fill this gap, we developed the TSPA tool for children and use it by caregivers or healthcare professionals. In this study, it is an initial development and partial psychometric properties. For content and design, the resulting interpretation was further developed into platform applications for easier access and widely used in the future.

## 2. Materials and Methods

The developmental research was aimed to develop the TSPA tool for children and psychometric properties of content validity, construct validity, internal consistency, and intra–rater reliability. This research received ethical approval, from the Associated Medical Sciences Research Ethical Committees, Chiang Mai University, code AMSEC-64EX-037.

### 2.1. Procedure

#### 2.1.1. Phase 1: Drafting a Questionnaire and Designing Result Interpreting for the TSPA Tool for Children

The purpose of this phase was to develop a simple questionnaire and create item to measure the behavioral response of children to sensory stimuli in everyday life and receive a report from the caregiver. It was based on the literature review about theoretical sensation, assessment of sensory processing patterns and the criteria of psychometric properties.

Previous study from the literature review found that the concept of existing tools had the purpose of measuring both atypical and typical people. For example, the tools by Jean Ayres [20,21] and Milleret et al. [22] aimed to measure sensory processing disorder, while those of Dunn and Brown [23,24] aimed to identify sensory processing patterns of typical persons. However, interpretation of sensory processing was limited to four patterns overall with these tools and they did not categorize each sensory domain. On the other hand, the TSPA tool for adolescents and adults developed more than 15 years by Srikhamjak et al. [15,16,17,18] aimed to measure typical persons by using simple sensory processing to interpret a variety of dimensions and categorize each sensory stimulus. aimed to measure typical persons by using simple sensory processing to interpret a variety of dimensions and categorize each sensory stimulus.

The TSPA tool for adolescents and adults has been continuously developed for simplicity in use since the original version developed in 2007 by Srikhamjak et al. In 2020, Pomngen et al. [16,17] reviewed the concept of the TSPA tool for adolescents and adults, finding that it is simple to use to cooperate with health providers and clients. In order to develop a new TSPA for children, the concept of the adult and adolescent versions of TSPA was adapted. Therefore, the first draft of a TSPA tool for children is divided into two parts (sensory preference and sensory arousal), each containing six sensory modalities. By requesting caregiver reports on the behavior of children in everyday life, we created items that are appropriate for measuring the children’s response to sensory stimuli. Rating frequency criteria and scoring items are all included. In addition, we designed a simple and easy-to-use output interpretation for the TSPA tool for children that shows sensory patterns.

#### 2.1.2. Phase 2: Examining Content Validity

We choose a content validity to examine was established using the index of item-objective congruence (IOC). This statistical procedure, developed by Rovinelli and Hambleton, 1997 [25], is best used in test development to assess content validity at the item development stage [26]. The content validity was performed by experts rating individual items and processes following guidelines and previous studies [27,28,29,30].

Determining qualification of five experts for content valid examine as follows (1) who specialize in sensory processing theory (2) who have experience using assessment instruments or measurements (3) experience in the field of research (4) have experience in the clinic of sensory processing in children at least five years.

We contact to expert in the field above mentioned and send the first draft of TSPA tool for children. In the IOC process, experts rated individual items based on whether they agree or disagree with the specific objectives. Individual experts rated using a 3-point scale as follow; 1 (a definite indicator of sensory preference and sensory arousal in each sensory modality), 0 (undecided), and −1 (not an indicator of sensory preference and sensory arousal in each sensory modality). In addition, giving suggestions for revised items.

IOC is calculated after expert ratings of individual items are completed. For content validity, IOC analysis took IOC values up to 0.80 to be considered acceptable [26] and removed if the item was the IOC value < 0.8. However, items with acceptable IOC values were revised if clarification was suggested by the expert.

After revising the TSPA tool for children of items that acceptable level was pilot testing in similarly caregivers. The pilot testing for checking is understandable and could be used by interviews and listening to feedback form caregivers between pilot tests. Therefore, the second draft of TSPA tool for children were used to collecting data for reliability and construct validity examine.

#### 2.1.3. Phase 3: Examining of Reliability and Construct Validity

##### Internal Consistency and Construct Validity

Sample size: Based on the location and population, there were 44,192 caregivers. The sample size was estimated by the calculation formula of Yamane and determined reliability to 95% or to have an error of 0.05. Definition: *n* = number of sample size, *N* = number of populations, *e* = error by followed guidelines [31,32].

$$n=\frac{N}{1+Ne^{2}}$$

A sample size of 396 was calculated. In factor analysis, a minimum sample size of at least 300 is recommended by Comrey and Lee [33]. Children aged 3–12 years are randomly sampled from schools in four districts in Chiang Mai and their caregivers at every class level to estimate the proportion of access samples.

Participant data were collected through multistage random sampling. In the first step, we randomly selected 30 schools from four districts in Chiang Mai, Thailand, and sent letters to the principals of those schools. We collected data from 16 schools and advertised our research in 16 schools. To participate in this study, parents or caregivers who have normal children aged 3–12 were invited. A previous study with access to participants and data collection [34] is the basis of this research.

Theses caregivers received the invitation letter includes information sheet explanation of the purpose and process of study and an information sheet. The purpose of this study was to collect data during the COVID-19 period and the social distancing rules that were in place at that time. The participant has the option of choosing response items on-site or online. Each caregiver filled out a consent form, provided demographic information, and completed the second draft of TSPA for children.

After caregivers have completed the TSPA tool for children in this phase, we collected the information in the first round of the internal consistency study. We selected the information according to the inclusion criteria for the data analysis. Construct validity was examined for the retention of items that were appropriate for factor analysis and removed items that did not meet criteria. The next step was to finalize the TSPA tool for children and to analyze the data by internal consistency.

##### Intra-Rater Reliability

After the data collection of the first round, we invite the caregiver to complete the TSPA tool for children twice (at 2 weeks intervals after the first round) to assess intra-rater reliability. Caregivers who participated were invited to enroll and the appointment date in the data collection. The caregivers completed the TSPA tool for children in the second round. Consequently, intra-rater reliability was analyzed based on first and second data from caregivers.

### 2.2. Data Analysis

All data analyses were performed using the IBM.SPSS. Statistics for Windows, Version 26.0.

Content validity was examined by experts who rated each item using a 3-point scale: 1 (a definite indicator of sensory preference and sensory arousal in each modality), 0 (undecided), and −1 (not an indicator of sensory preference and sensory arousal in each modality). The IOC value of ≥ 0.80 was considered an acceptable level and represented high content validity [27,28,29,30].

Construct validity was examined by factor analysis that indicated whether it was appropriate for each of the senses as individual subscales in the analysis. Factor structures of sensory preferences and sensory arousal in each sense were examined by principal components analysis with varimax rotation. The Kaiser criterion (Eigenvalues > 1) and proportion of total variance explained the criteria implemented for the number of factors to be extracted. Items that had poor factor loadings (<0.40) or were cross-loaded on two or more factors were removed [35,36,37].

Internal consistency reliability was established by using Cronbach’s alpha coefficient, with criteria using the standard detailed by Arikanto, 1992: < 0.4 = poor, 0.41–0.70 = acceptable, 0.71–1.00 = excellent [38].

Inter-rater reliability was examined using the intraclass correlation coefficient (ICC), with interpretation using standards detailed by Cicchetti, 1999: < 0.40 poor, 0.40–0.59 acceptable, 0.60–0.74 good, 0.75–1.0, excellent [39].

## 3. Results

### 3.1. Phase 1: Drafting the TSPA for the Children Questionnaire

The first drafted TSPA tool for children was a caregiver observation-report questionnaire designed to evaluate the sensory processing patterns of normal children and divide into two parts: part 1—sensory preferences and part 2—sensory arousal. Each part comprised six sensory modalities in sight, sound, smell and taste, touch, vestibular, and proprioceptive. The first draft of the TSPA for children consisted of 99 items (part 1: 51 items and part 2: 48 items).

This tool determined caregivers to give information by choosing answers following the frequency (never, rarely, sometimes, often, always) of children’s behavior response to sensory stimuli. The tool measured the frequency of response to sensory stimuli using the Likert scale from 1–5 and determined scoring for each item (1 = never, 2 = rarely, 3 = sometimes, 4 = often, 5 = always) in the sensory preference part. While sensory arousal part (1 = never, 2 = rarely, 3 = sometimes, 4 = often, 5 = always) in items with high arousal and (5 = never, 4 = rarely, 3 = sometimes, 2 = often, 1 = always) in items with low arousal.

For designed interpret results by determining score each item and then calculating percentages and present them for simple to use as a radar chart.

The radar chart presents sensory patterns in variety and individual. The radar chart/plot can show characteristics and properties that can present both lines of sensory preferences and arousal. A radar chart can show together a line/plot which is easy to use, and maybe a line shown in preference and arousal similar level or different level. Moreover, this pattern displays the integration of sensory preferences and arousal of each sense. For instance, in the Figure above, when the radar chart/plot showed low arousal and high preference that presents the tendency to the response of the seeking person. While however, if it displays high arousal and low preference that presents the tendency to the response of the sensitive person.

### 3.2. Phase 2: Examining Content Validity

The content validity of the tool was examined by five experts (a specialized pediatric occupational therapist, pediatric occupational therapy lecturer, pediatrician, family physician, and club president caregiver of autistic people). The index of the IOC was 0.8–1.00 The TSPA second draft had 93 accepted items consisting of part 1: 46 sensory preference items and part 2: 47 sensory arousal items. It also had 33 piloted caregivers who found the language clear and were able to collect data.

### 3.3. Phase 3: Reliability and Construct Validity

In following ethical research approval, the participants comprised 444 caregivers of normal children aged 3–12 years and who were interested in obtaining information. However, 30 caregivers did not meet the criteria and were excluded from the analysis because of caregiver incomplete response items and caregivers who have atypical children. Therefore, a total of 414 sample data of normal children were inclusion, consisted of age range, with 132 (31.9%) being 3–6 years old, and 282 (68.1%) 7–12 years. In this case, 196 (47.3%) were male and 218 (52.7%) females. Caregivers who complete items comprising 368 (88.9%) parents, 20 relatives (4.8 %), 2 nannies (0.5%) and 24 others (5.8 %). Data collected from analysis are shown as follows.

#### 3.3.1. Internal Consistency

Cronbach’s alpha coefficient was used to examine the internal consistency of the TSPA tool for children second draft, and the results are shown in Table 1. Cronbach’s α coefficient showed that the TSPA was 0.92 (excellent) for overall items in part 1: sensory preference, and 0.80 (excellent) in part 2: sensory arousal.

#### 3.3.2. Construct Validity

Factor analysis using the principal component was carried out for each subscale. The Kaiser-Mayer-Olkin (KMO) measure of sampling adequacy and Bartlett’s Test of Sphericity were examined, and items were removed if their sampling adequacy was below 0.5. Factor extraction was based on examination of the Kaiser criterion of eigenvalues over 1.00. Additional consideration was given to the theoretical interpretation of the factor. Factors were not retained if they had fewer than three items. Items were deleted if they had factor loadings of less than 0.4, or were loaded on a factor that was not interpretable.

Part 1: Sensory preferences consisted of six modalities (sight, sound, smell and taste, touch, vestibular, and proprioceptive). By following the data analysis shown in Table 2, each modality was found to have a KMO range of 0.717–0.855, chi-square of 438.611–853.440 and significance < 0.001, which was appropriate for factor analysis. Sight comprised 6 items (factor loading rang of 0.666–0.804), with only one item removed. Sound comprised 6 items (factor loading rang of 0.527–0.758), with two removed. Smell and taste comprised 6 items, with two removed. Touch comprised 5 items (factor loading rang of 0.533–0.710), with two removed. Vestibular comprised 7 items (factor loading rang of 0.600–0.732), with none removed from the second daft. Proprioception comprised 5 items (factor loading rang of 0.604–0.719), with two removed. Therefore, part 1: sensory preference contained 35 items, and Table 2 presents the factor loading of items were retained in each sense.

Part 2: Sensory arousal consisted of six modalities (sight, sound, smell and taste, touch, vestibular, and proprioception). By following the data analysis shown in Table 3, each modality yielded a KMO each modality yielded a KMO range of 0.660–0.822, chi-square of 313.129–682.784 and significance <0.001, which was appropriate for factor analysis. Sight comprised 5 items (factor loading rang of 0.569–0.679), with two items removed. Sound comprised 5 items (factor loading rang of 0.619–0.767), with two removed. Smell and taste comprised 6 items (factor loading rang of 0.503–0.741), with two removed. Touch comprised 6 items (factor loading rang of 0.449–0.732), with four removed. Vestibular comprised 4 items (factor loading rang of 0.569–0.791), with three removed. Proprioception comprised 4 items (factor loading rang of 0.433–0.692), with three removed. Therefore, part 2: sensory arousal contained 30 items, and Table 3 presents the factor loading of items were retained in each sense. By following the factor analysis, the TSPA questionnaire were contained total 65 items, comprise of 35 items of sensory preferences part and 30 items of sensory arousal part. Next, internal consistency for final and intra-rater reliability was examined.

#### 3.3.3. Internal Consistency of Items in the Final Factor Analysis

After the items were removed from the factor analysis in the TSPA questionnaire, the consistency of response to those of part 1 and 2 were examined again. The results showed Cronbach’s α. coefficient of 0.92 (excellent) and 0.81 (excellent) in part 1 and 2, respectively. Internal consistency of Cronbach’s α. coefficient is shown in Table 4.

#### 3.3.4. Intra-Rater Reliability

Intra-rater reliability was examined in the data of 40 normal children aged 3–12 years. The caregivers of these 40 children completed the TSPA tool for children twice to examine consistency between the ratings provided by the same rater. The intraclass correlation coefficient for part 1: sensory preference with 35 items was 0.74 (good) and part 2: sensory preference with 30 items was 0.79 (excellent). Intra-rater reliability was examined in the data of 40 children aged 3–12 years, as shown in Table 5.

## 4. Discussion

This study aimed to develop and examine psychometric properties of the TSPA tool: caregiver-version for children aged 3–12 years. The results showed that both parts of the tool had acceptable content validity, construct validity, internal consistency, intra-rater reliability. Only proprioceptive sense in part 2: sensory arousal had a lowest score for Cronbach’s α coefficient (Table 4), as found in previous studies [15,16,17,18], in which proprioceptive sense in sensory arousal part had the lower than others. This might be due to the fact that this sensory modality differed from other senses. Firstly, there are two types of proprioceptive: conscious and non-conscious. In non-conscious proprioceptive, impulses that arise from this type of sensation are delayed to the cerebellum rather than to the cerebral cortex [40,41]. This naturally causes proprioceptive sense to be rarely noticed. Secondary, in the clinical aspect, the behavior of children who actively seek proprioceptive sense such as hitting, pushing and rough play are often associated with displays of aggression [42]. This might lead to the perspective of caregivers being subjective and bias. These conditions and previous study demonstrate no single measure of proprioceptive due to the complexity [43]. Therefore, to decrease bias and more strong information may considered proprioceptive sense integrate with others assessment. It is important that the caregiver understands the purpose of this tool and how the results are used. It should be explained that sensory patterns are reflections of who we are, and not a pathology that needs to be remedied. Once understood, sensory patterns of the child open the door to an enriched life, which leads to the authors’ suggestion of cooperation between caregiver and pediatric professions in sharing more data.

**Figure 1:**
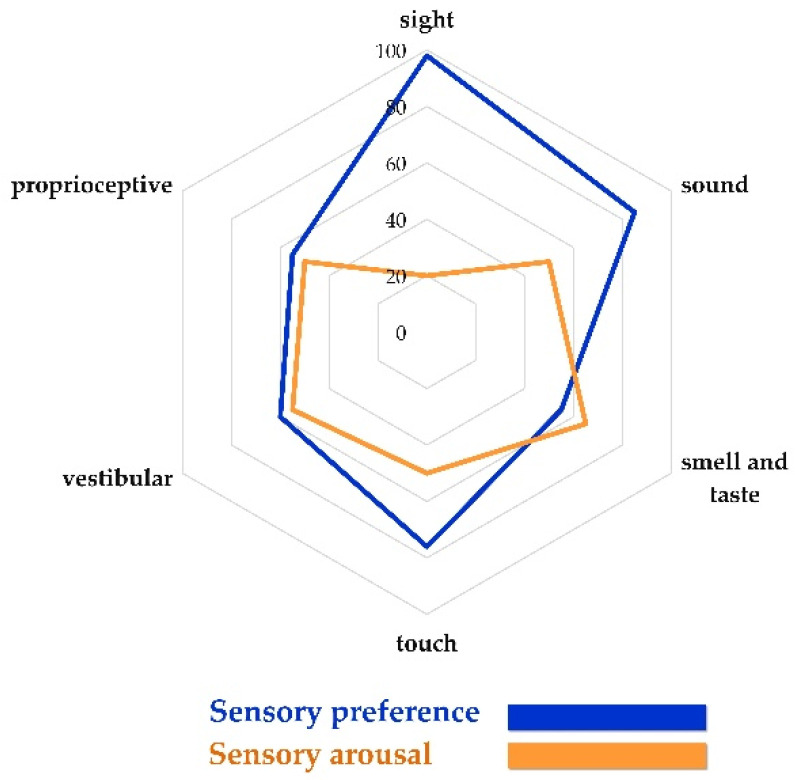
Radar chart of TSPA interpretation.

However, although further verification of the TSPA tool for children is needed, this new tool showed much potential and differing points from previous tools. Firstly, the radar chart was used to present interpretation of the sensory processing pattern that provides a brief picture for easily understanding the individual. A line that intersects the web can easily be perceived and interpreted as the tendency between high and low scores. Secondly, by integrating the level of preferences and arousal in each sense, information for deeply understanding the behavior of children can be achieved in a variety of dimensions. For example, Figure 1. shows a radar chart of sensory processing patterns, which reports a particular preference in sight and sound. While result integrating preferences and arousal in each sense which report sight has high preference and low arousal than the others. This is similar to Dunn’s sensory seeking pattern, but that sought only sight sense and not senses overall, which is different to the TSPA and previous tools. The TSPA tool can interpret a variety of sensory processing patterns, while Dunn’s pattern is limited to four. Once family caregivers and pediatric professionals including healthcare professionals have insight into the variety of sensory patterns, the child is offered a way to prefer what it needs to do in growing up in its own way.

This TSPA tool for children enhances caregiver’s understanding of their children. Firstly, we knew the level of sensory preference and sensory arousal to describe the behavior response and meaningful activity of their children. Which result interpretation by radar chart help caregiver understand how much preference and arousal each sensory modality of individual children. Information on sensory patterns can help the caregiver’s understanding of sensory needs with impact on children’s satisfying experiences led to the quality of life and relationship in the family. Importantly, understand the identity of each child and their unique set of talents. It is important to recognize acceptance in children and not force them, but rather support them in choosing their way. In-home and community contexts, caregivers can use results to promote children’s health. To build intrinsic motivation to participate in daily routines in self-care, eating, and sleeping, we need to pay attention to sensory preferences. When children demonstrate healthy behavior, they can be rewarded based on sensory preference. Sensory arousal is used in sensory-based environments to create safe and comfortable areas for children. There is a relationship between high sensory arousal and stress, blood pressure, and mental health [44]. Similarly, sleep problems are frequently associated with children who are easily over-aroused and are tactile sensitive. There is a link between sensory hypersensitivity and lower sleeping quality among children [45,46,47].

By integrating preferences and arousal into the radar chart, healthcare professionals can deeply understand the interpretation of the results. Radar charts revealed many patterns, including sensory preferences outside sensory arousal, sensory arousal outside preference, and sensory preferences balanced with arousal. It may be helpful for healthcare professionals to obtain an understanding of the sensory needs of individual children and the services that are provided by the client’s center. To increase the effectiveness of health programs, the sensory preferences of children should be taken into account. It is possible to obstruct effective sensory arousal while providing an appropriate amount of stimulation. Furthermore, the result can be applied to describe children not only from a neuroscience perspective but from an educational and social perspective as well. According to a previous study [48], sensory processing patterns are strongly related to persons with introversion and extroversion. In this tool, caregivers would respond to items, and the results were calculated as a percentage and presented as a radar chart. Nevertheless, in this paper-based study, only items from the TSPA tool for children were examined. It is the first development of a TSPA tool for children that is developed on the platform application for the next steps. Future applications can evaluate both caregivers and healthcare professionals by the program can automatically result and strategies ready to use.

Finally, it will be feasible to use the TSPA tool for children on the telehealth in the future, as the process of collecting data continued during the COVID-19 pandemic. The hybrid approach was used for collecting data, both onsite and over distances via online. From the hybrid approach, the TSPA tool was found to be available online, and its benefits are convenience for the caregiver, saving time and costs, and safety in a crisis. Similar study previously predicted that the hybrid approach will become the norm in the future, with telehealth being used to support families from a distance [49]. Implication for future may be developing this tool on a technological platform, from which telehealth supports healthcare services for children in the future.

However, this is first to studies TSPA tool for children in Thai context and studies some psychometric property. For more strong potential of this tools and benefit for widely others children is need to continuously developed. Thus, further research should be study criteria cut score, convergent and discriminant validity and if possible, may be examine for cross cultural children in others context.

## 5. Conclusions

In summary, the development and examination of the TSPA tool for children were satisfactory. This assessment has been content validity and construct validity and internal consistency and intra-rater reliability for measuring sensory processing patterns of normal children aged 3–12 years. To pediatric profession and family caregivers can cooperate use this tool to understand and promote for their children. It is recommended that users should consider this point (proprioceptive sense) when using TSPA for children, as well as other information from the child’s medical history. In future research, the proprioceptive of sensory arousal could be revised and increased examined, along with predictive validity, discriminative validity, and cut score criteria. Implication, to appropriate with world changing and increase healthcare services accessed the researcher may be to develop this TSPA on technology platform in the future.

## Figures and Tables

**Table 1:** Internal consistency estimates of the TSPA before factor analysis (*n* = 414).

Part 1: Sensory Preference	No.of Items	α.			Part 2: Sensory Arousal	No.of Items	α.		
		3–6 Years	7–12 Years	Overall			3–6 Years	7–12 Years	Overall
Sight	7	0.81	0.77	0.79	Sight	7	0.69	0.57	0.61
Sound	8	0.73	0.77	0.78	Sound	7	0.61	0.55	0.57
Smell and Taste	8	0.76	0.74	0.74	Smell and Taste	8	0.74	0.71	0.72
Touch	8	0.69	0.65	0.68	Touch	10	0.77	0.72	0.73
Vestibular	7	0.83	0.78	0.81	Vestibular	7	0.52	0.53	0.53
Proprioceptive	8	0.73	0.73	0.73	Proprioceptive	8	0.22	0.20	0.20
Total	46	0.91	0.92	0.92	Total	47	0.81	0.79	0.80

**Table 2:** Factor loading of part 1: sensory preference.

Part 1: Sensory Preference							
Subscale	Items	Factor	KMO	Bartlett’s Test		% of Variance	Eigenvalues
				Chi-Square	Sig.		
Sight	SPsight5	0.804	0.855	832.626	<0.001.	47.02	3.291
	SPsight2	0.803					
	SPsight1	0.732					
	SPsight4	0.728					
	SPsight6	0.697					
	SPsight7	0.666					
Sound	SPsound2	0.758	0.786	849.329	<0.001	40.22	3.218
	SPsound4	0.748					
	SPsound6	0.675					
	SPsound1	0.661					
	SPsound3	0.565					
	SPsound8	0.527					
Smell and Taste	SPsmell&taste8	0.727	0.726	736.198	<0.001	36.403	2.912
	SPsmell&taste6	0.710					
	SPsmell&taste7	0.669					
	SPsmell&taste2	0.620					
	SPsmell&taste1	0.589					
	SPsmell&taste5	0.529					
Touch	SPtouch8	0.710	0.751	438.611	<0.001	31.928	2.554
	SPtouch2	0.669					
	SPtouch5	0.566					
	SPtouch6	0.520					
	SPtouch3	0.505					
Vestibular	SPvestibular6	0.732	0.810	853.440	<0.001	47.470	3.323
	SPvestibular2	0.724					
	SPvestibular3	0.716					
	SPvestibular4	0.695					
	SPvestibular5	0.689					
	SPvestibular7	0.658					
	SPvestibular1	0.600					
Proprioceptive	SPproprio1	0.719	0.717	700.833	<0.001	35.444	2.836
	SPproprio5	0.660					
	SPproprio3	0.636					
	SPproprio2	0.631					
	SPproprio6	0.604					

**Table 3:** Factor loading of sensory arousal in each modality.

Part 2: Sensory Arousal							
Subscale	Items	Factor	KMO	Bartlett’s Test		% of Variance	Eigenvalues
				Chi-Square	Sig.		
Sight	SAsight2	0.679	0.660	374.184	<0.001	31.308	2.192
	SAsight6	0.675					
	SAsight5	0.631					
	SAsight1	0.630					
	SAsight7	0.569					
Sound	SAsound5	0.767	0.758	435.991	<0.001	35.502	2.485
	SAsound7	0.705					
	SAsound3	0.699					
	SAsound6	0.696					
	SAsound4	0.619					
Smell and Taste	SAsmell&taste6	0.741	0.770	682.784	<0.001	35.355	2.828
	SAsmell&taste4	0.695					
	SAsmell&taste7	0.678					
	SAsmell&taste3	0.677					
	SAsmell&taste5	0.644					
	SAsmell&taste8	0.503					
Touch	SAtouch6	0.732	0.822	659.627	<0.001	31.173	3.117
	SAtouch5	0.692					
	SAtouch8	0.672					
	SAtouch4	0.653					
	SAtouch7	0.537					
	SAtouch9	0.449					
Vestibular	SAves4	0.791	0.738	405.672	<0.001	34.115	2.388
	SAves5	0.757					
	SAvestibular1	0.666					
	SAvestibular7	0.569					
Proprioceptive	SAproprio2	0.692	0.677	313.129	<0.001	27.060	2.165
	SAproprio1	0.611					
	SAproprio5	0.520					
	SAproprio7	0.433					

**Table 4:** Internal consistency after items were removed from the factor analysis.

Part 1: Sensory Preference	No. of Item	α.			Part 2: Sensory Arousal	No. of Item	α.		
		3–6 Years	7–12 Years	Overall			3–6 Years	7–12 Years	Overall
Sight	6	0.81	0.82	0.83	Sight	5	0.71	0.62	0.65
Sound	6	0.72	0.75	0.76	Sound	5	0.76	0.72	0.74
Smell and Taste	6	0.77	0.73	0.74	Smell and Taste	6	0.76	0.73	0.74
Touch	5	0.61	0.59	0.62	Touch	6	0.76	0.70	0.72
Vestibular	7	0.78	0.77	0.81	Vestibular	4	0.68	0.70	0.69
Proprioceptive	5	0.70	0.70	0.64	Proprioceptive	4	0.42	0.50	0.47
Total	35	0.90	0.91	0.92	Total	30	0.83	0.80	0.81

**Table 5:** Intra-rater reliability and intraclass correlation coefficient of the TSPA.

Part 1: Sensory Preference	No. of Items	ICC	Part 2: Sensory Arousal	No. of Items	ICC
Sight	6	0.79	Sight	5	0.75
Sound	6	0.64	Sound	5	0.77
Smell and Taste	6	0.55	Smell and Taste	6	0.63
Touch	5	0.65	Touch	6	0.56
Vestibular	7	0.66	Vestibular	4	0.78
Proprioceptive	5	0.67	Proprioceptive	4	0.64
Total	35	0.74	Total	30	0.79

## Data Availability

The data in this study are available on request from the corresponding author.
